# Location-Specific Association Between Cerebral Microbleeds and Arterial Pulsatility

**DOI:** 10.3389/fneur.2019.01012

**Published:** 2019-09-18

**Authors:** Kun-Hsien Chou, Pei-Ning Wang, Li-Ning Peng, Li-Kuo Liu, Wei-Ju Lee, Liang-Kung Chen, Ching-Po Lin, Chih-Ping Chung

**Affiliations:** ^1^Institute of Neuroscience, National Yang Ming University, Taipei, Taiwan; ^2^Brain Research Center, National Yang Ming University, Taipei, Taiwan; ^3^Department of Neurology in School of Medicine, National Yang Ming University, Taipei, Taiwan; ^4^Aging and Health Research Center, National Yang Ming University, Taipei, Taiwan; ^5^Department of Neurology, Taipei Veterans General Hospital, Taipei, Taiwan; ^6^Center for Geriatric and Gerontology, Taipei Veterans General Hospital, Taipei, Taiwan; ^7^Department of Family Medicine, Taipei Veterans General Hospital Yuanshan Branch, Taipei, Taiwan

**Keywords:** cerebral microbleeds, pulsatility index, arterial pulsatility, cerebral amyloid angiopathy, cerebral small vessel disease

## Abstract

**Objective:** Increased arterial pulsatility index (API), usually representative of distal vascular resistance, have been linked to cerebral small vessel disease. However, their relationship with cerebral microbleeds (CMBs) is less well-studied. The present study aimed to evaluate the relationship between CMBs and API.

**Methods:** We cross-sectionally evaluated participants from a non-clinical stroke, non-demented community-based population. APIs of cervical internal carotid and vertebral arteries were measured by ultrasonography. CMBs were assessed by susceptibility-weighted-imaging on 3T magnetic resonance imaging (MRI). Subjects were classified according to CMB locations: deep/infratentorial (DI) or strictly lobar (SL) CMB groups. DI-CMB group also included subjects with simultaneous lobar CMBs.

**Results:** Of the 681 subjects [62.2 (8.4) years, 43.5% men] included, CMBs were found in 92 (13.5%) subjects: 57 (8.4%) with DI-CMB and 35 (5.1%) with SL-CMB. The results showed that CMB location influenced their association with API. DI-CMB was significantly associated with elevated API of internal carotid arteries (β = 0.031; 95% confidence interval = 0.002–0.059; *P* = 0.03), while SL-CMB was significantly associated with elevated API of vertebral arteries (β = 0.050; 95% confidence interval = 0.006–0.094; *P* = 0.025) in multivariate analyses adjusting for age, sex, cardiovascular risk factors, white matter hyperintensities (WMH), and lacunes.

**Conclusion:** Our study again emphasizes (1) the association between API and cerebral small vessel disease and (2) the pathogenic differences between DI- and SL-CMBs. Our results lead to the postulation that in the presence of CMBs without clinical dysfunction yet, insidious small vascular disorders might already occur with corresponding topography.

## Introduction

Cerebral microbleeds (CMBs) are well-demarcated, hypointense, rounded lesions on magnetic susceptibility-sensitive sequences of magnetic resonance imaging (MRI) and their histopathology shows hemosiderin-laden macrophages (1, 2). In addition to white matter hyperintensities (WMH) and lacunes, CMB is another common imaging marker of cerebral small vessel diseases (CSVDs) (3).

The arterial pulsatility index (API) is calculated using Gosling's equation as [(peak systolic flow velocity (PSV)—end-diastolic flow velocity (EDV))/mean flow velocity] and is usually measured by Doppler ultrasound (4). An elevated API is commonly associated with increased downstream flow resistance in conditions, such as severe stenosis or occlusion of distal vessels (4, 5). Correlating to this concept, studies have found positive associations between an elevated API in large arteries supplying cerebral circulation and the presence of WMH, a consequence of cerebral microvascular pathologies (6). Recently, an alternative theory emerged to explain the relationship between increased API and WMH; elevated cervical and cerebral APIs might originate from systemic or central arterial stiffness, e.g., aorta stiffness, which then transmits a pulsatile force into the brain resulting in injury of the cerebral microvessels (6–8).

Unlike WMH, scant studies have evaluated the relationship between API and CMBs. To elucidate the pathophysiology underlying the relationship between elevated API and CSVDs, we will need more studies on other CSVD features in addition to WMH. CMBs are initially clinically silent and accumulate with aging or progression of disease such as stroke or dementia (1, 2). As a result, CMBs are mostly detected in the later stages of disease, in patients who have had a stroke or suffer from dementia. Most previous CMB research has been studied in patients with stroke or dementia, in which results might be confounded by several comorbidities. Information regarding CMBs before symptom onset is required for elucidating the independent effect and early pathophysiology of CMBs. The present study aimed to explore the relationship between CMBs and API of cervical arteries for cerebral anterior and posterior circulation, e.g., the internal carotid artery (ICA) and vertebral artery (VA), in a non-clinical stroke, non-demented community-based population, presumed to be at a pre-clinical or prodromal stage of disease. Since CMBs occurring in different locations have different manifestations, they are deemed to have a distinct pathophysiology (1, 2, 9–11). We hypothesized that the association between API and CMBs also depended on the location of CMBs. We also assessed other CSVDs (WMH and lacunes) by MRI and systemic arterial stiffness with pulse pressure (PP) measurement, and took these factors into account during analysis. These results may offer new insights into both the early pathophysiology of CMBs and the underlying mechanisms mediating the association between elevated API and CSVDs.

## Methods and Materials

### Study Population and General Assessment

The I-Lan Longitudinal Aging Study (ILAS) is a community-based aging cohort study in the I-Lan County of Taiwan that aimed to evaluate the interrelationship between geriatric syndromes and brain structural abnormalities. Furthermore, the ILAS looked to explore predictors or associated factors for future disability, dementia, or mortality in the geriatric population in the prodromal or early stages of disease. The study protocol has been previously described in detail (12). Inhabitants, who met the study inclusion criteria, were randomly sampled from the household registration data of the county's government. Selected inhabitants were invited to participate by mail or telephone. The inclusion criteria were as follows: (1) having no plans for moving out of I-Lan County in the near future, and (2) being 50 years of age or older. Subjects who met any one of the following conditions were excluded: (1) unable to adequately communicate with the interviewer, (2) having a disabled status (modified Rankin Scale > 2), (3) limited life expectancy (<6 months) due to major illness, and (4) currently institutionalized. Additionally, subjects that had any contraindication to an MRI such as metal implants or known neuropsychiatric diseases such as dementia, stroke, or brain tumor by self-report, neuropsychological assessment or brain MRI were excluded from the study. The study was approved by the Institutional Review Board of the National Yang Ming University, Taipei, Taiwan. All participants provided informed consent, and the study was conducted in accordance with the relevant ethical guidelines and regulations. Participants' data were given a de-identified number after collection. All collected data (paper and electronic data) were stored in the encrypted files.

A questionnaire was used to collect data regarding the demographics and medical history of the study subjects. The height, weight, and resting blood pressure (BP; from brachial artery) of the subjects were measured. PP was calculated as [systolic BP (SBP)—diastolic BP (DBP)]. Fasting serum lipid levels (total cholesterol, low density lipoprotein, high density lipoprotein, and triglyceride), sugar level, HgbA1c, and blood urea nitrogen and creatinine levels were determined by a chemical analyzer (ADVIA 1900, Siemens, Malvern, PA, USA). All participants received a face-to-face neuropsychological examination administered by trained interviewers. Global cognitive performance was assessed by the mini-mental state examination (MMSE). We defined global cognitive impairment as an MMSE score <24 in well-educated persons (education ≥6 years) or <14 in less-educated persons (education <6 years) (13). To elucidate the early pathophysiology in the preclinical stage of disease, subjects with global cognitive impairment were excluded from the study.

### Definitions of Cardiovascular Risk Factors

The presence of cardiovascular risk factors including smoking cigarettes was determined by patient history or laboratory investigation. Hypertension was defined as patients self-reporting use of antihypertensive medication or as a measurement of SBP ≥ 140 mmHg or DBP ≥ 90 mmHg. Diabetes mellitus (DM) was defined as patients self-reporting DM treatment or a measurement of HgbA1c 6.5%. Hyperlipidemia was recorded if patients self-reported the use of a statin agent or a total blood cholesterol level ≥240 mg/dL. Chronic kidney disease (CKD) was defined as an estimated glomerular filtration rate≤ 60 mL/min/1.73 m.

### Brain MRI Acquisition and CMBs Assessment

All of the participants underwent a brain MRI study at National Yang-Ming University, Taipei, Taiwan. Images were acquired on a 3T Siemens MRI scanner (Siemens Magnetom Tim Trio, Erlangen, Germany) with a 12-channel head coil. An axial T2-weighted fluid attenuated inversion recovery (FLAIR) multi-shot turbo spin echo sequence with BLADE technique was acquired with the following parameters: repetition time (TR) = 9,000 ms, echo time (TE) = 143 ms, inversion time = 2,500 ms, flip angle = 130 degree, number of excitation = 1, echo train length = 35, matrix size = 320*320, field of view (FOV) = 220*220 mm^2^, 63 slices, bandwidth = 252 Hz/Px, voxel size = 0.69*0.69*2.0 mm^3^ without inter-slice gap, and an acquisition time = 7 min and 41 s. Three-dimensional susceptibility-weighted imaging (SWI) was used to identify CMBs. A three dimensional SWI sequence was acquired with the following parameters: TR = 28 ms, TE = 21 ms, flip angle = 15 degree, matrix size = 256*224, FOV = 256*224 mm^2^, 88 slices, bandwidth = 120 Hz/Px, voxel size = 1.0*1.0*2.0 mm^3^ without inter-slice gap, and acquisition time = 9 min and 13 s.

**Figure 1:**
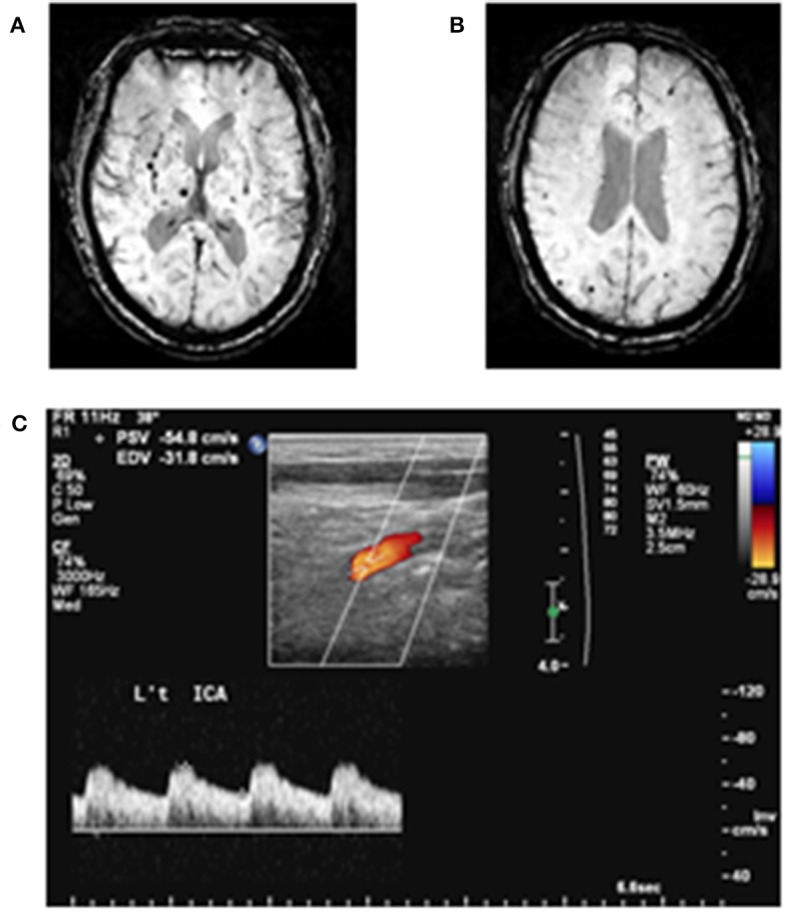
Examples of cerebral microbleeds and arterial pulsatility index measurement. **(A)** Deep cerebral microbleeds and **(B)** lobar cerebral microbleeds are demonstrated. **(C)** Doppler spectrum of internal carotid artery. Arterial pulsatility index is defined as [(peak systolic flow velocity (PSV)—end-diastolic flow velocity (EDV))/mean flow velocity].

CMBs were defined as small, rounded or circular, well-defined hypointense lesions within the brain parenchyma with clear margins that were ≦10 mm in size on the SWI image (3, 14). Microbleed mimics such as vessels, calcification, partial volume, air-bone interfaces, and hemorrhages within or adjacent to an infarct were carefully excluded. We distinguished calcification by viewing T1-weighted MRI. CMB mimics showing low signal intensity on a T1-weight MRI were regarded as calcification. We used the Microbleed Anatomical Rating Scale to measure the presence, amount, and topographic distribution of CMBs in each subject, which has been reported to have a good intra-rater and inter-rater reliability (15). The microbleeds were classified according to whether they were located in the deep, infratentorial, or lobar regions. Lobar topography was determined according to Stark and Bradley (16), and included the cortical and subcortical regions (including subcortical *U* fibers). The lobar CMBs were assessed in the fontal, parietal, temporal, and occipital regions. The deep regions included the basal ganglia (BG), thalamus, internal capsule, external capsule, corpus callosum, and deep/periventricular white matter. The infratentorial regions included the brainstem and cerebellum. DPWM was defined as white matter adjacent to or within ~10 mm of the lateral ventricular margin. Images were displayed and viewed using the MRIcro software (version 1.40, Chris Rorden's MRIcro) by one neurologist (Dr. Chung) who was blinded to the clinical data during CMB assessment and analyses. CMBs in 20 randomly sampled subjects' images were evaluated again at a separate time, and the intra-rater was calculated (*k* = 0.83; 95% confidence interval: 0.79–0.90). We also re-assessed CMBs in 25 randomly sampled subjects' images by Dr. Chung and a well-trained assistant (Mr. Ching-Sern Yong). The inter-rater k was 0.82 (95% confidence interval: 0.79–0.88) (Figure 1).

Subjects were classified into three groups: (1) no-CMB group: absence of CMB; (2) deep or infratentorial (DI)-CMB group: presence of CMBs in deep or infratentorial regions (DI-CMB group also included subjects with CMBs located in both deep/infratentorial and lobar regions); and (3) strictly lobar (SL)-CMB group: presence of CMBs exclusively in lobar regions.

The other manifestations of CSVDs, the numbers of lacunes, and the severity of WMH were also recorded in every subject by FLAIR-T2-weighted MRI. Lacunes are small CSF-containing cavities, ≤ 15 mm in diameter, located in the deep gray or white matter with adjacent white matter hyperintensity (3). The severity of WMH was rated by the modified Fazekas scale (17). The Fazekas scale scores are: 0 = WMH, 1 = WMH, 2 = moderate WMH, and 3 = severe WMH.

### Neck Color-Coded Duplex Ultrasonography

All of the subjects underwent ultrasound imaging of the bilateral cervical arteries in longitudinal and cross-sectional projections using an instrument (GE LOGIQ 400 PRO; GE, Cleveland, OH, USA) equipped with a high-resolution broadband width linear array transducer by 1 technician who was unaware of the clinical characteristics of the subjects. Measurements of blood flow velocity were additionally taken on a separate visit in 20 random-sampled subjects, and the intra-rater *k* = 0.80 (95% confidence interval 0.77–0.89) (Figure 1).

Blood flow velocity readings were obtained, after at least 5 min rest in the supine with position with the patient's head on a pillow, from the bilateral common carotid artery, ICA and VA, including PSV and EDV. We calculated mean flow velocity and API from measured PSV and EDV. Measurement regions used for analyses were 1–1.5 cm distal to the carotid bifurcation for ICAs and the cervical V1 segment for VAs. Subjects detected with having >50% focal stenosis or occlusion in the cervical arteries were excluded from the study (5).

### Statistical Analyses

Analyses were performed with SPSS software (version 22.0; IBM, USA). All data are presented as means [standard deviation (SD)] for continuous variables and numbers (percentage) for discrete variables. Group comparisons were made using the analysis of variance (ANOVA) test. Bonferroni correction was applied for pairwise group comparisons. Hemodynamics of each side's ICA and VA were compared. When appropriate, a chi-square (χ2) test or Fisher's exact test was performed for categorical variables. Multivariate linear regression analyses were used to evaluate the association between API of the cervical arteries and CMBs. Mean API of bilateral ICAs and VAs were used as variables. Adjusting factors included age, sex, cardiovascular risk factors (hypertension, DM, dyslipidemia, cigarette smoking, and CKD), WMH, lacunes, DBP, or PP. A *p*-value of < 0.05 was deemed statistically significant.

## Results

From the initial group of 748 subjects, 36 subjects with cognitive impairment and 9 subjects with an incidentally found brain tumor following MRI were excluded. There were also 16 subjects with imaging artifacts due to head motion and 6 subjects with cervical arteries >50% stenosis. Thus, a total of 681 subjects were included in the study with a mean age of 62.2 (SD = 8.4; range = 50.0–87.7 years). Two-hundred and ninety-six of the subjects were men (43.5%). The prevalence of cardiovascular risk factors in the population was as follows: hypertension, 37.2%; DM, 14.0%; hyperlipidemia, 5.4%; cigarette smoking, 14.2%; and CKD, 1.2%.

CMBs were found in 92 (13.5%) of the subjects. Among them, most (59, 8.7%) had only 1 lesion, 16 (2.3%) subjects had 2 CMBs, 10 (1.4%) had 3–5 CMBs, and 7 (0.9%) had >5 CMBs. Most CMBs, both in terms of prevalence and number, were found in deep brain regions (49 subjects, 7.2%). Infratentorial and lobar CMBs were found in 13 subjects (1.9%), and 48 subjects (7.0%), respectively. 6 (0.8%) subjects had cerebellar CMB(s). There was no subject with cerebellar CMBs having simultaneous CMBs exclusively located in lobar regions. There were 57 subjects (8.4%) with DI-CMB, and 35 subjects (5.1%) with SL-CMB. Among 35 subjects with SL-CMB, 12 (34.3%) had CMB located in frontal lobe, 8 (22.9%) parietal lobe, 9 (25.7%) temporal lobe and 8 (22.9%) occipital lobe. Assessment of other CSVDs showed that 28 (4.1%) subjects had more than one lacune and 95 (14.0%) subjects had moderate to severe WMH (Fazekas scale score of 2–3).

### Comparisons Among no-CMB, DI-CMB, and SL-CMB Groups

Table 1 shows the results of comparisons. Among several demographics, age and the prevalence of hypertension significantly differed between the 3 groups. *Post-hoc* analyses showed that the no-CMB group was significantly younger than the DI-CMB and SL-CMB groups, respectively. The prevalence of hypertension and hyperlipidemia were higher in the DI-CMB compared with the no-CMB and SL-CMB groups, but the difference was only statistically significant for the prevalence of hypertension.

**Table 1:** Comparisons of clinical, imaging and hemodynamic characteristics.

	**No-CMB (*n* = 589)**	**DI-CMB (*n* = 57)**	**SL-CMB (*n* = 35)**	***p***
**CLINICAL/IMAGING CHARACTERISTICS**				
Age, years, mean (SD)	61.6 (8.0)	66.9 (10.2)	65.1 (8.8)	<0.001
Sex, men, *n* (%)	251 (42.6)	28 (49.1)	17 (48.6)	0.525
Hypertension, *n* (%)	211 (35.8)	31 (54.4)	11 (31.4)	0.017
Diabetes mellitus, *n* (%)	77 (13.1)	11 (19.3)	7 (20.0)	0.246
Hyperlipidemia, *n* (%)	28 (4.8)	7 (12.3)	2 (5.7)	0.057
Cigarette smoking, *n* (%)	148 (25.1)	15 (26.3)	8 (22.9)	0.922
Chronic kidney disease, *n* (%)	6 (1.0)	2 (3.5)	0	0.200
HgbA1c, %, mean (SD)	6.0 (0.8)	6.1 (1.1)	6.0 (0.8)	0.419
Hypertensive medicines, *n* (%)	100 (17.0)	11 (19.3)	3 (8.6)	0.374
Moderate/severe WMH, *n* (%)	52 (9.1)	32 (56.1)	11 (34.4)	<0.001
Lacunes > 1, *n* (%)	10 (1.7)	16 (28.0)	2 (5.7)	<0.001
**HEMODYNAMIC CHARACTERISTICS**				
SBP, mmHg, mean (SD)	127.9 (15.8)	132.8 (18.2)	129.1 (22.1)	0.095
DBP, mmHg, mean (SD)	78.8 (12.8)	83.2 (13.2)	76.5 (10.8)	0.023
HR, per minute, mean (SD)	72.0 (10.2)	72.8 (10.4)	70.8 (14.1)	0.682
PP, mmHg, mean (SD)	49.1 (10.1)	49.7 (11.7)	52.6 (15.1)	0.165
**Right ICA, cm/s, mean (SD)**				
Peak systolic velocity	65.8 (6.5)	64.8 (6.2)	66.4 (6.3)	0.427
End diastolic velocity	31.2 (4.3)	29.1 (5.3)	30.7 (4.7)	0.002
**Left ICA, cm/s, mean (SD)**				
Peak systolic velocity	66.6 (6.4)	65.3 (5.8)	66.7 (7.2)	0.333
End diastolic velocity	31.9 (4.4)	29.4 (4.9)	30.8 (4.6)	<0.001
**Right VA, cm/s, mean (SD)**				
Peak systolic velocity	34.7 (4.7)	33.8 (4.0)	34.5 (5.0)	0.439
End diastolic velocity	14.7 (2.8)	13.9 (2.5)	13.9 (1.8)	0.026
**Left VA, cm/s, mean (SD)**				
Peak systolic velocity	35.2 (5.0)	34.3 (4.7)	33.3 (4.2)	0.049
End diastolic velocity	15.2 (2.9)	14.1 (2.4)	13.4 (2.2)	<0.001
**Pulsatility index, mean (SD)**				
Right ICA	0.81 (0.11)	0.88 (0.16)	0.84 (0.13)	<0.001
Left ICA	0.80 (0.11)	0.88 (0.14)	0.84 (0.13)	<0.001
Right VA	0.94 (0.14)	0.98 (0.16)	1.00 (0.14)	0.031
Left VA	0.92 (0.14)	0.98 (0.15)	1.00 (0.17)	0.001

*CMB, cerebral microbleeds; DI, deep or infratentorial; SL, strictly lobar; WMH, white matter hyperintensities; SBP, systolic blood pressure; DBP, diastolic blood pressure; HR, heart rate; PP, pulse pressure; ICA, internal carotid artery; VA, vertebral artery*.

The prevalence of moderate to severe WMH and lacunes were significantly different among three groups (Table 1). DI-CMB and SL-CMB groups had higher prevalence of moderate to severe WMH and lacunes > 1 than no-CMB; DI-CMB group had the highest prevalence of moderate to severe WMH (56.1%) and lacunes > 1 (28%) among three groups.

SBP and DBP were both higher in the DI-CMB group compared with the other 2 groups, however, only DBP showed statistical significance (Table 1). *Post-hoc* analyses showed that DBP in the DI-CMP group was significantly higher than in the no-CMB and SL-CMP groups, respectively. PP, representative of systemic arterial stiffness, was similar between the 3 groups.

Though DBP was higher in the DI-CMB group and similar PP was observed between the 3 groups, cervical arterial EDVs were lower in the CMB groups compared with the no-CMB group (Table 1). *Post-hoc* analyses showed that EDVs in bilateral ICAs were significantly lower in the DI-CMB group than in the no-CMB group. With regards to VAs, both DI-CMB and SL-CMB had significantly lower EDVs than the no-CMB group in *post-hoc* analyses.

### CMB Groups Had Higher APIs Compared With no-CMB Group

**Figure 2:**
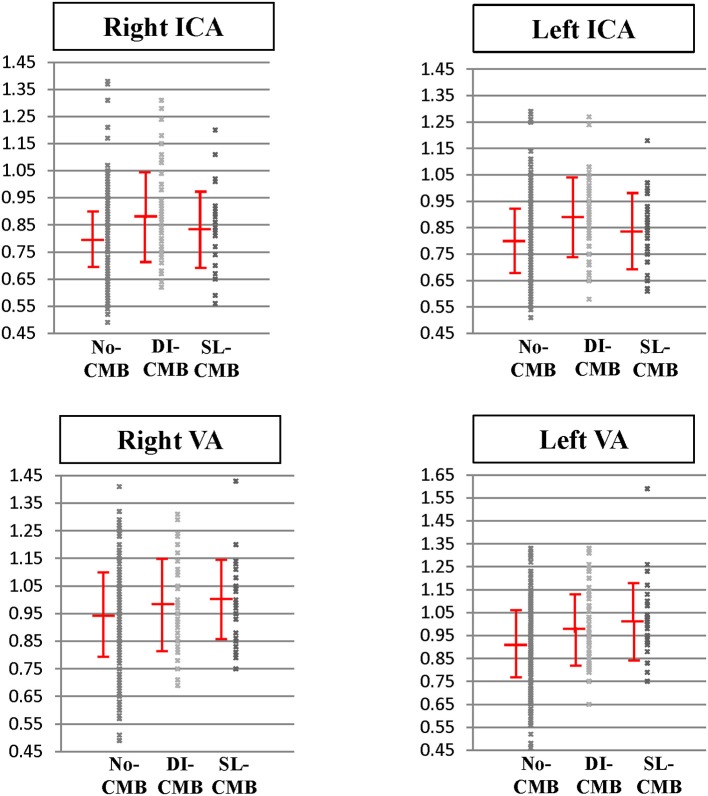
Pulsatility index of cervical arteries in subjects with no cerebral microbleed, deep/infratentorial cerebral microbleeds and strictly-lobar cerebral microbleeds. Pulsatility index of internal carotid arteries and vertebral arteries in three groups are expressed as scatter plots with mean and standard deviation bars. DI-CMB, deep/infratentorial cerebral microbleeds; ICA, internal carotid artery; No-CMB, no-cerebral microbleed; SL-CMB, strictly-lobar cerebral microbleeds; VA, vertebral artery.

Table 1 and Figure 2 reveal APIs of cervical arteries of the 3 groups. We performed Shapiro-Wilk test to evaluate the normality of API in each CMB group. The results showed that APIs in both ICA and VA were distributed normally in each group (DI-CMB group: ICA *p* = 0.082; VA *p* = 0.759; SL-CMBs group: ICA *p* = 0.176; VA *p* = 0.118). The DI-CMB group had the highest APIs in ICAs between the 3 groups and *Post-hoc* analyses showed that APIs of bilateral ICAs in the DI-CMB group were significantly higher than in the no-CMB group. With regards to the posterior cervical arteries, the SL-CMB group had the highest APIs of VAs between the three groups. *Post-hoc* analyses showed that APIs of left VA in the DI-CMB and SL-CMB groups were both significantly higher than in the no-CMB group.

### Associations Between CMBs and API

Table 2 shows the correlation analyses between the numbers of CMBs and API of cervical arteries. Mean API of bilateral VAs and ICAs were used for analyses. The results showed that the numbers of total CMBs, DI-CMBs, and SL-CMBs were significantly associated with APIs of VA and ICA, respectively.

**Table 2:** Correlation between numbers of cerebral microbleeds and arterial pulsatility.

	**Total CMB numbers**	**Deep or infratentorial CMB numbers**	**Lobar CMB numbers**
**PI of ICAs**			
Spearman correlation	0.183	0.154	0.127
*p* value	<0.001	<0.001	0.001
**PI of VAs**			
Spearman correlation	0.128	0.093	0.107
*p* value	0.001	0.016	0.006

*CMB, cerebral microbleeds; PI, pulsatility index; ICA, internal carotid artery; VA, vertebral artery*.

### Independent Location-Specific Associations Between CMBs and API

Tables 3, 4 show the results of multivariate linear regression analyses. Dependent variables were the mean API of bilateral ICAs (Table 3) and of bilateral VAs (Table 4), respectively. The results after adjusting for confounding factors showed that the location of CMBs influenced their association with API; DI-CMB was significantly associated with elevated API of ICA while SL-CMB was significantly associated with elevated API of VA.

**Table 3:** Multivariate analyses of the association between cerebral microbleeds and pulsatility of internal carotid artery.

	**B coefficient**	**95% confidence interval**	***p***
**DI-CMB, yes vs. no**			
Model 1	0.048	0.023–0.074	<0.001
Model 2	0.047	0.022–0.073	<0.001
Model 3	0.031	0.002–0.059	0.031
Model 4	0.047	0.022–0.073	<0.001
Model 5	0.048	0.022–0.073	<0.001
**SL-CMB, yes vs. no**			
Model 1	0.022	−0.009–0.054	0.159
Model 2	0.021	−0.011–0.052	0.193
Model 3	0.014	−0.019–0.047	0.404

*Model 1 adjusted for age and sex; Model 2 adjusted for age, sex, vascular risk factors (hypertension, diabetes mellitus, hyperlipidemia, cigarette smoking and chronic kidney disease); Model 3 adjusted for age, sex, vascular risk factors, white matter hyperintensities and lacunes; Model 4 adjusted for age, sex, vascular risk factors, white matter hyperintensities, lacunes and diastolic blood pressure; Model 5 adjusted for age, sex, vascular risk factors, white matter hyperintensities, lacunes and pulse pressure*.

*CMB, cerebral microbleeds; DI, deep or infratentorial; SL, strictly lobar*.

**Table 4:** Multivariate analyses of the association between cerebral microbleeds and pulsatility of vertebral artery.

	**B coefficient**	**95% confidence interval**	***p***
**DI-CMB, yes vs. no**			
Model 1	0.021	−0.013–0.054	0.223
Model 2	0.020	−0.013–0.054	0.233
Model 3	0.001	−0.036–0.038	0.942
**SL-CMB, yes vs. no**			
Model 1	0.048	0.007–0.089	0.023
Model 2	0.048	0.006–0.090	0.024
Model 3	0.050	0.006–0.094	0.025
Model 4	0.049	0.0007–0.091	0.021
Model 5	0.046	0.004–0.087	0.032

*Model 1 adjusted for age and sex; Model 2 adjusted for age, sex, vascular risk factors (hypertension, diabetes mellitus, hyperlipidemia, cigarette smoking and chronic kidney disease); Model 3 adjusted for age, sex, vascular risk factors, white matter hyperintensities and lacunes; Model 4 adjusted for age, sex, vascular risk factors, white matter hyperintensities, lacunes and diastolic blood pressure; Model 5 adjusted for age, sex, vascular risk factors, white matter hyperintensities, lacunes and pulse pressure*.

*CMB, cerebral microbleeds; DI, deep or infratentorial; SL, strictly lobar*.

Since increased API of the cervical arteries might also represent systemic arterial stiffness, we further performed multivariate analyses adjusting for DBP or PP, parameters of systemic arterial stiffness (Table 4). The results showed no attenuated associations between elevated cervical APIs and CMBs, indicating that systemic arterial stiffness might play a minor role in these associations.

## Discussion

The present study revealed an association between CMBs and APIs in a non-clinical stroke, non-demented population. Our results also showed that different location of both CMBs and cervical arteries had different patterns of association; DI-CMB was significantly correlated with elevated API of the cervical arteries supplying the cerebral anterior circulation, e.g., ICA, while SL-CMB was significantly correlated with elevated API of the cervical arteries supplying the cerebral posterior circulation e.g., VA. The associations were independent of age, sex, cardiovascular risk factors, and other CSVDs (WMH and lacunes).

More and more evidence suggests that high cerebral API might be involved in the pathophysiology of CSVDs (6). These studies mostly used WMH to represent CSVD burden, and it is unclear whether high API is also related to other CSVDs such as CMBs. In addition, most studies only measured API in anterior cervical or cerebral arteries such as ICA or middle cerebral arteries. The present study measured API of both anterior and posterior cervical arteries. We are the first to show an association between API and CMBs and find a topographic differentiation in this association.

Studies have observed that characteristics differ between DI- and SL-CMBs (1, 2, 9–11, 18–20). DI-CMBs are associated with the presence of hypertension, elevated BP, and other features of hypertensive vasculopathy, while SL-CMBs are associated with features of cerebral amyloid angiopathy (CAA) such as APOEε4 genotype and amyloid accumulation on positron emission tomography (PET) (21). Hypertensive vasculopathy, such as lipohyalinosis or arteriosclerosis, usually involves deep penetrating arteries perpendicular to the middle cerebral arteries (21); CAA is known to affect vessels over the caudal brain regions (22–24). Since APIs of the cervical arteries may result from downstream flow resistance due to intracranial vascular abnormalities, our results might reflect this difference of underlying vascular pathologies and suggest that, in the presence of CMBs, insidious small vascular disorders have already occurred with corresponding topography in the non-clinical stroke, non-demented population.

It is not surprising to see a relationship between elevated API and DI-CMBs since DI-CMBs are known to be associated with hypertension, which will cause arteriosclerosis and arterial stiffness in both systemic and cerebral arteries. The present study took markers of systemic arterial stiffness, PP, into analyses and found that the significance of association between elevated API and DI-CMBs were not attenuated. This result suggests that elevated API shown in subjects with DI-CMBs is more likely due to distal vascular resistance instead of systemic or central arterial stiffness. The presence of DI-CMB might be an indicator that cerebral arteries have been affected before the occurrence of significant clinical symptoms and systemic arterial stiffness.

Previous studies suggest that CAA has a preference for posterior cerebral microvessels involvement (22–24). Amyloid accumulation, elevated cerebral API, impaired cerebral vasoreactivity and WMH have been shown more significantly in posterior circulation in patients with CAA (22–26). Our results also showed a similar topographic preference of elevated API in SL-CMB and again support SL-CMB as a marker of CAA.

The brain, like the kidney, has the unusual physiological characteristic of continuous, passive high-volume perfusion with very low vascular resistance, which makes the brain vasculature highly susceptible to pressure and flow fluctuations. Previous studies have provided evidence that aorta stiffness might cause cognitive impairment and CSVD, particularly WMH, via pulsatile force transmitted by the cervical arteries reflected by elevated API (6–8). The present study did not assess aortic stiffness, therefore, could not evaluate the role of aortic stiffness in the relationship between cervical API and CMBs. Our results also could not exclude that, in addition to reflective of downstream vasculopathy, elevated cervical API is a causality of downstream cerebral microvascular disorders by transmitting a pulsatile force into susceptive brain circulation in DI- and SL-CMBs.

The strengths of the present study include the community-based setting and considerable sample size. The present study population was non-clinical stroke, non-demented subjects, which presume to be at the early phase of CSVDs. Therefore, our results might be applied to the early pathophysiology of CSVDs. Furthermore, the present study took other common CSVDs, WMH and lacunes, and locations of both CMBs and measured cervical arteries into consideration. Since age and cardiovascular risk factors are also related to elevated API, the present study has adjusted for age, sex, cardiovascular risk factors and other markers of systemic stiffness in multivariate analyses. We were able to show the independent relationship between CMBs and API.

However, this study has limitations. First, the present study was a cross-sectional study. Second, we did not take VA hypoplasia into consideration due to a lack of VA diameter recordings. We took the API of both the left and right VA for group comparisons and the results indeed revealed that the API of the left VA, the less prevalent side of VA hypoplasia (5), was more significantly associated with CMBs. Therefore, in multivariate analyses, we used the mean API of the bilateral VAs instead of the individual VA, in an attempt to attenuate this structural factor. Third, we would need to include other parameters such as pulse-wave velocity, central BP, and other central hemodynamics to determine the role of systemic or central arterial stiffness in the associations between CMBs and elevated cervical APIs. Fourth, we used voxel size = 1.0*1.0*2.0 mm^3^ without inter-slice gap instead of 1 mm^3^ or the other better resolutions since the size of CMBs is defined as 2–10 mm^3^. Lastly, we measured API in the cervical rather than intracranial arteries, which could avoid the loss of >20% data due to insufficient acoustic windows and incorrect intracranial arterial flow velocity measurements due to the assumed angle of insonation that occur with transcranial Doppler ultrasound (5). Nevertheless, whether intracranial arteries, more representative of microvascular resistance, have similar hemodynamic changes with cervical arteries shown in the present study would need further studies to validate our postulation about the relationship between API and CMBs.

In conclusion, the present study revealed an association between elevated cervical API and CMBs in non-clinical stroke and non-demented community-based population. DI-CMB was significantly associated with elevated API of the ICA and SL-CMB was significantly associated with elevated API of the VA, independent of age, sex, cardiovascular risk factors, WMH, and lacunes. Our results again emphasize the pathogenic differences between DI- and SL-CMBs. The presence of DI- and SL-CMBs, even without obvious clinical symptoms, may be a marker of cerebral microvascular disorders of distinct topographic distribution and etiologies.

## Data Availability Statement

All datasets generated for this study are included in the manuscript/supplementary files.

## Ethics Statement

The studies involving human participants were reviewed and approved by National Yang Ming University IRB. The patients/participants provided their written informed consent to participate in this study.

## Author Contributions

C-PC: had full access to all of the data in the study and takes responsibility for the integrity of the data and the accuracy of the data analysis. K-HC, L-KC, C-PL, P-NW, and C-PC: study concept and design, acquisition, analysis, and interpretation of data. K-HC and C-PL: MRI technical and material support. L-NP, W-JL, and L-KL: study subjects data collection and study execution. All authors have read and approve of the final version of the manuscript.

### Conflict of Interest

The authors declare that the research was conducted in the absence of any commercial or financial relationships that could be construed as a potential conflict of interest.
